# Biocontrol of bacterial wilt disease in tomato using *Bacillus subtilis* strain R31

**DOI:** 10.3389/fmicb.2023.1281381

**Published:** 2023-09-28

**Authors:** Yunhao Sun, Yutong Su, Zhen Meng, Jie Zhang, Li Zheng, Shuang Miao, Di Qin, Yulan Ruan, Yanhui Wu, Lina Xiong, Xun Yan, Zhangyong Dong, Ping Cheng, Mingwei Shao, Guohui Yu

**Affiliations:** ^1^Innovative Institute for Plant Health, Zhongkai University of Agriculture and Engineering, Guangzhou, China; ^2^Key Laboratory of Green Prevention and Control on Fruits and Vegetables in South China, Ministry of Agriculture and Rural Affairs, Guangzhou, China; ^3^Guangdong University Key Laboratory for Sustainable Control of Fruit and Vegetable Diseases and Pests, Guangzhou, China; ^4^College of Agriculture and Biology, Zhongkai University of Agriculture and Engineering, Guangzhou, China; ^5^College of Resources and Environment, Zhongkai University of Agriculture and Engineering, Guangzhou, China; ^6^School of Life Sciences, Sun Yat-sen University, Guangzhou, China

**Keywords:** bacterial wilt disease, *Bacillus subtilis*, *Ralstonia solanacearum*, biological control, lipopeptides, microbiome

## Abstract

Bacterial wilt disease caused by *Ralstonia solanacearum* is a widespread, severe plant disease. Tomato (*Solanum lycopersicum*), one of the most important vegetable crops worldwide, is particularly susceptible to this disease. Biological control offers numerous advantages, making it a highly favorable approach for managing bacterial wilt. In this study, the results demonstrate that treatment with the biological control strain *Bacillus subtilis* R31 significantly reduced the incidence of tomato bacterial wilt. In addition, R31 directly inhibits the growth of *R. solanacearum*, and lipopeptides play an important role in this effect. The results also show that R31 can stably colonize the rhizosphere soil and root tissues of tomato plants for a long time, reduce the *R. solanacearum* population in the rhizosphere soil, and alter the microbial community that interacts with *R. solanacearum*. This study provides an important theoretical basis for elucidating the mechanism of *B. subtilis* as a biological control agent against bacterial wilt and lays the foundation for the optimization and promotion of other agents such as R31.

## Introduction

Bacterial wilt disease caused by *Ralstonia solanacearum* is a widespread, severe plant disease, affecting more than 200 plant species of more than 53 botanical families. This disease is especially prevalent in tropical, subtropical, and warm-temperature regions, where it significantly reduces the yields of various crops such as tomato (*Solanum lycopersicum*), pepper (*Capsicum annuum*), potato (*Solanum tuberosum*), peanut (*Arachis hypogaea*), and banana (*Musa* spp.; Genin, 2010). Due to its destructive nature, *R. solanacearum* has gained recognition as a leading pathogenic agent and is extensively studied for its role in causing bacterial wilt (Li et al., 2017; Liu et al., 2017; Sun et al., 2017, 2019; Ye et al., 2019). Tomato, one of the most important vegetable crops worldwide (with annual yields of approximately 160 million tons), is particularly susceptible to this disease (Khan et al., 2021). Unfortunately, the common practice of continuous cropping has aggravated this problem, leading to the prevalence of soil-borne diseases such as tomato bacterial wilt, which poses a significant threat to the tomato production industry on a global scale (Garcia-Estrada et al., 2023).

In the absence of host plants, *R. solanacearum* can survive in soil or water for a long time (Ascarrunz et al., 2011). Attempts to combat this bacterium through plant breeding, field sanitation, crop rotation, and the use of bactericides have not been successful. Many of these methods also pose significant issues, such as environmental pollution and the generation of pesticide residues. These concerns have raised doubts about the safety of agricultural products.

To promote environmentally friendly, sustainable agricultural practices, the control of bacterial wilt through biological means has become an increasing focus of study (Chen et al., 2019; Elsayed et al., 2019). Biological control offers numerous advantages over chemical control, including reduced pollution, the preservation of natural enemies of pathogens, decreased risk of developing resistance, enhanced safety for humans and animals, and improved environmental protection. Furthermore, biological control enables integrated pest management strategies and aligns with the principles of organic agriculture, making it a highly favorable approach for managing bacterial wilt.

Several recent studies have demonstrated the potential of using antagonistic bacteria for the biological control of bacterial wilt disease. For instance, the suppressive effects of certain antagonistic bacteria on *R. solanacearum* have been reported (Khokhani et al., 2017). Additionally, Fu et al. demonstrated the effectiveness of the biocontrol of tomato bacterial wilt by foliar spray application of a strain of endophytic *Bacillus* sp. (Fu et al., 2020). Several studies have focused on the antimicrobial lipopeptides produced by biological control strains, which have garnered significant interest (Ben Abdallah et al., 2015; Chen et al., 2019). Lipopeptides exhibit a wide range of activities against bacteria, fungi, viruses, mycoplasma, and tumor cells (Romano et al., 2011; Lam et al., 2021). Lipopeptides are also known for their stability, low toxicity to humans and animals, and environmentally friendly properties (Cochrane and Vederas, 2016). Furthermore, lipopeptide antibiotics can enhance the colonization of biological control strains in plant roots and induce systemic resistance in the host (Lam et al., 2021). The importance of lipopeptides in suppressing plant diseases is widely recognized, making them potentially valuable tools as biocontrol agents against plant diseases.

We previously isolated *Bacillus subtilis* strain R31 from the endophytic community of *Dendrobium officinale* leaves (Li et al., 2011; Zhang et al., 2016; Cheng et al., 2017; Zhang et al., 2017; Li et al., 2021). R31 has broad-spectrum antagonistic activity against plant pathogenic fungi (Li et al., 2011; Zhang et al., 2016; Cheng et al., 2017; Zhang et al., 2017; Li et al., 2021). During our years of research and treatment using biological control agents in banana, we determined that the application of R31 has good control effects against banana wilt disease (Li et al., 2011; Zhang et al., 2016; Cheng et al., 2017; Zhang et al., 2017; Li et al., 2021). The commercial microbial agent product “Diyixi” and the microbial fertilizer “Dingwei,” which contain R31 as their core component, were registered in the China Rural Agriculture Bureau and have been used in various parts of Guangdong Province, China. These agents have been applied for the prevention and control of various crop diseases.

In this study, we analyzed the effects of R31 against tomato bacterial wilt and determined that the early application of *B. subtilis* R31 significantly reduces the incidence of this disease. Moreover, R31 directly inhibits the growth of *R. solanacearum*, in which lipopeptides secreted by R31 play an important role. We also confirmed that R31 can stably colonize the rhizosphere soil and roots of tomato plants for a long time, reduce *R. solanacearum* contents in the rhizosphere soil, and alter the interactions of *R. solanacearum*–related microorganisms in the microbial community. These findings provide an important theoretical basis for elucidating the mechanism of action of *B. subtilis* in the biological control of bacterial wilt and lay the foundation for optimizing and promoting the use of this type of agent.

## Methods

### Plant materials and analysis of plant disease symptoms

The tomato plant variety used in this study was *Solanum lycopersicum* L. cv. “Money Maker.” The seeds were soaked in water, heated to 60°C for 2 h, placed in a Petri dish lined with wet filter paper, and incubated in a climate chamber at 25°C–29°C and 60% relative humidity for 4–7 days for germination. Once the seeds had germinated, seedlings were transferred to 32-hole pots containing sterilized mixed nutrient soil (nutrient soil to vermiculite at a 1:1 weight ratio). The seedlings were watered with 10-fold diluted Hoagland nutrient solution (Qingdao Haibo Biotechnology). When the seedlings had developed four true leaves, they were transplanted into planting bags (16 × 14 cm) filled with a mixture of yellow clay soil and vegetable planting soil (3:1 weight ratio). The plants were fertilized once a week with 10-fold diluted Hoagland nutrient solution. The strain inoculation experiment was conducted when the plants had reached the six-leaf stage.

Following inoculation with *R. solanacearum*, the disease severity index of the plants was observed at 7 days post inoculation, with scores of 0 (no wilting symptoms, healthy), 1 (0%–25% wilting leaves), 2 (25%–50% wilting leaves), 3 (50%–75% wilting leaves), and 4 (75%–100% wilting leaves). The disease incidence, disease severity, and biocontrol efficiency were calculated based on the disease severity index. The data obtained from the calculations were analyzed using one-way analysis of variance (ANOVA) and multiple comparisons (Duncan’s test) using SPSS 21.0.


$$\mathrm{Disease}\;\mathrm{incidence}=\left(\frac{\mathrm{Total}\;\mathrm{number}\;\mathrm{of}\;\mathrm{diseased}\;\mathrm{plants}}{\mathrm{Total}\;\mathrm{number}\;\mathrm{of}\;\mathrm{tested}\;\mathrm{plants}}\right)\times 100\%$$

$$\begin{array}{l} \mathrm{Disease}\;\mathrm{severity}=\\ \frac{\sum \left(\begin{array}{l} \mathrm{Number}\;\mathrm{of}\;\mathrm{plants}\;\mathrm{with}\;\mathrm{corresponding}\;\mathrm{disease}\;\mathrm{index}\\ \times \mathrm{disease}\;\mathrm{severity} \end{array}\right)}{\begin{array}{l} \mathrm{Total}\;\mathrm{number}\;\mathrm{of}\;\mathrm{tested}\;\mathrm{plants}\times \\ \mathrm{highest}\;\mathrm{disease}\;\mathrm{level} \end{array}}\times 100\% \end{array}$$

$$\mathrm{Biocontrol}\;\mathrm{efficiency}=\frac{{DS}_{CK}-{DS}_{T}}{{DS}_{CK}}\times 100\%$$

### Inoculation of plants and plate confrontation experiments

*Bacillus subtilis* strains R31 and 168 (Itaya, 2022) were preserved in our laboratory. The R31 strain was activated on an LB agar plate and cultured at 37°C for 12 h. A single colony was selected, inoculated into 5 mL of LB liquid medium, and cultured at 37°C for 12–14 h. A 50-μL aliquot of inoculum was transferred into 5 mL of LB medium and cultured at 37°C for 4 h. Subsequently, 12 mL of inoculum was transferred into 200 mL of nutrient broth (NB) medium (Sangare et al., 2014) and cultured at 37°C for 48 h to obtain the fermentation broth. *Ralstonia solanacearum* strain GMI1000 was preserved in our laboratory. GMI1000 was activated on a casamino acid-peptone-glucose (CPG) agar plate (Jung et al., 2017) and cultured at 30°C for 72 h. A single colony was selected, inoculated into 150 mL of CPG liquid medium, and cultured at 30°C for 16–18 h with shaking at 180 rpm to obtain the *R. solanacearum* fermentation broth.

For inoculation of the R31 strain, each root of a tomato seedling with six leaves was inoculated with 50 mL of *B. subtilis* R31 fermentation broth (1 × 10^6^ CFU/mL, 1 × 10^7^ CFU/mL, and 1 × 10^8^ CFU/mL, respectively) using the root immersion method (Shao et al., 2023). For inoculation of the GMI1000 strain, the root irrigation method was used (Genin, 2010). One side of each tomato seedling was wounded with sterilized scissors, and 50 mL of 1 × 10^7^ colony-forming units (CFUs)/mL *R. solanacearum* was inoculated at the wound site. An equal volume of sterile water was used as the control treatment.

For the strain confrontation experiment using agar medium mixed with bacteria, 0.5 mL fermentation broth containing 1 × 10^8^ CFU/mL *R. solanacearum* was mixed with 20 mL of cooled CPG agar medium that had not yet solidified. After incubating the plates at 30°C for 0, 12, or 14 h, sterile filter paper disks were placed on the medium and 10 μL of 1 × 10^8^ CFU/mL *B. subtilis* R31 fermentation broth was dropped onto the disks. The plates were incubated at 30°C for 3 days.

For the strain confrontation experiment using agar medium spread with bacteria, 100 μL fermentation broth containing 1 × 10^8^ CFU/mL *R. solanacearum* was spread evenly on the surface of CPG agar medium. A 10-μL aliquot of 1 × 10^8^ CFU/mL *B. subtilis* R31 fermentation broth was dropped onto the plate. Alternatively, 10 μL of 1 × 10^8^ CFU/mL R31 bacterial suspension that was prepared by washing the bacterial cells with sterile water twice and resuspending in sterile water was dropped onto the plate. The plates were incubated at 30°C for 3 to 4 days.

### Antibacterial experiment using crude lipopeptides from *Bacillus subtilis* R31

The gene clusters responsible for the biosynthesis of the secondary metabolites of *B. subtilis* R31 were predicted using the sequenced whole-genome data of strain R31 (Li et al., 2021) and antiSMASH software (Kai et al., 2021). Crude lipopeptides were extracted from the fermentation broth of *B. subtilis* R31 using the acid precipitation method (Chen et al., 2017). The fermentation broth was centrifuged at 4°C at 10,000 rcf for 10 min to remove the bacterial cells. After adjusting the pH to 2.0, the sample was incubated at 4°C for overnight precipitation. The suspension containing the precipitate was centrifuged at 4°C and 10,000 rcf for 10 min. The supernatant was discarded and the pellet combined with methanol for extraction; centrifugation and resuspension of the pellet in methanol were repeated three times. The solution was concentrated by rotary evaporation, and the concentrate was divided into two parts. One part was dissolved in 0.01 M phosphate-buffered saline (PBS, pH 7.2), resulting in a crude lipopeptide PBS solution for the antibacterial experiments; the other part was dissolved in chromatography-grade methanol for identification.

Liquid chromatography–mass spectrometry (LC-MS) was used to identify crude lipopeptides. The high-performance liquid chromatography conditions were as follows: flow rate of 1.0 mL/min and detection wavelength of 200–400 nm. The MS conditions were as follows: an electrospray ionization source was used, the capillary voltage was 4.5 KV, desolvation gas flow rate was 5.0 L/min, atomizer pressure was 0.8 Bar, and scanning range was 100–2,000 *m/z*.

When using agar culture medium to detect the antibacterial effect of crude lipopeptide extracts on *R. solanacearum*, 100 μL of 10^6^ CFU/mL *R. solanacearum* suspension was evenly spread onto CPG agar medium. Sterile Oxford cups were placed on the plate, and 100 μL crude lipopeptide extract was added to each cup. An equal volume of PBS was added as the control group. The culture was incubated at 30°C for 4 days.

To detect the effect of crude lipopeptide extracts on *R. solanacearum* biofilm, 1,980 μL of liquid CPG medium was added to a 24-well culture plate and mixed with 10 μL of 10^8^ CFU/mL *R. solanacearum* suspension. The experimental group was combined with 10 μL crude lipopeptide extract, and the control group was combined with phosphate buffer. After 48 and 72 h of culture, the amount of *R. solanacearum* biofilm formation was quantitatively measured by crystal violet staining. Briefly, the bacterial suspension was removed (creating a small hole), 1% (w/v) crystal violet was added through the hole, and the sample was allowed to be stained for 20 min. Each hole was washed with deionized water until the hole was colorless, and the crystal violet fixed in the biofilm was eluted using 33% (v/v) glacial acetic acid. Finally, the absorbance at 570 nm of each eluant was measured.

### Quantification of microbial titer, H_2_O_2_, and NO contents

To accurately isolate and detect the quantity of R31 in the samples, strain R31 carrying a rifampicin resistance cassette was used for soil and plant inoculation. Strain cultivation and inoculation were performed as described above. When collecting rhizosphere soil samples, 5 g of tomato roots (keeping the soil close to the root surface) was collected, placed in 200 mL of PBS, and incubated at 30°C with shaking at 180 rpm for 30 min. After removing the roots, the soil suspension was centrifuged at 8,000 rcf for 10 min at 30°C. The supernatant was removed, and 1 g of soil pellet was transferred to 9 mL of sterile water. The sample was vortexed at maximum speed for 10 min. Each sample was diluted six times in a 10-fold concentration gradient. A 100-μL aliquot of each concentration of sample was placed on a plate containing 300 μg/mL rifampicin. The plates were incubated at 37°C for 24 h before counting the colonies.

To collect root tissue samples, 1 g of tomato root tissues was collected, incubated in 75% (v/v) ethanol for 30 s followed by 2.5% (w/v) sodium hypochlorite for 60 s for surface sterilization, and washed four times with sterile water. Each sample was placed in a sterilized mortar, and sterile quartz sand was added for grinding. After grinding thoroughly, 9 mL of sterile water was added, and the sample was transferred to a 15-mL centrifuge tube. The sample was vortexed at maximum speed for 1 min and diluted six times in a 10-fold concentration gradient. A 100-μL aliquot of each concentration of sample was placed on a plate containing 300 μg/mL rifampicin. The plates were incubated at 37°C for 24 h before counting. To collect plant stem samples, a 1-cm stem sample was cut from the plant at 3 cm above the ground. The surface of the sample was disinfected by immersion in 75% (v/v) ethanol for 60 s, followed by immersion in 2.5% (w/v) sodium hypochlorite for 90 s. The other steps were the same as described for root sample collection and treatment.

To accurately detect the titer of GMI1000 in the samples, a fluorescence quantitative PCR system was used. The GMI1000 gene *RipAK* (Sun et al., 2017) was used as the detection indicator, and the NCBI Primer BLAST tool was used to design qPCR primers (forward primer 5′-CAATTGCTTCGCTCCTTCC-3′; reverse primer 5′-GCAAGCAGATCGCAAGTTC-3′; product size: 92 bp). Genomic DNA was extracted from 2 mL of *R. solanacearum* suspension, and the 5 × TA/Blunt Zero Cloning Mix system (Vazyme, China) was used to clone the gene in a plasmid for the production of quantitative standard curves. Using the above method to obtain soil samples, DNA was extracted from 0.3 g soil samples according to the instructions of the HiPure Soil DNA Mini Kit (D3142-03). Following qPCR amplification, the titer of *R. solanacearum* in the soil was calculated based on the Ct value.

To detect NO and H_2_O_2_ contents in roots, root samples were collected from tomato plants at specific time points and placed in a 2 mL grinding tube. After quickly added 0.1 M PBS (pH 7.2, pre-cooled at 4°C in advance), the roots were ground in a homogenizer. The ratio of root tissue to PBS was 1:9 (i.e., 0.2 g of root tissue was added to 1.8 mL of PBS). A 2-mL aliquot of homogenate was centrifuged at 4,000 rcf at 4°C for 10 min. The supernatant was transferred to a clean centrifuge tube and stored at 4°C for later use. The NO and H_2_O_2_ contents in the root tissue were determined using an H_2_O_2_ detection kit (S0038) and an NO detection kit (S0021S) according to the manufacturer’s instructions (Shanghai Biyuntian Biotechnology Co., Ltd.).

### Analysis of rhizosphere microorganisms

All rhizosphere samples were collected as described above and used to extract soil DNA. The *16S* rDNA V3-V4 region (GeneDenovo Biotechnology Co., Ltd.; forward primer 5′-CCTACGGRRBGASCAGKVRVGAAT-3′; reverse primer 5′-GGACTACNVGGGTWTCTAATCC-3′) was subjected to PCR amplification. The amplification products were sent to GeneDenovo Biotechnology Co., Ltd. for high-throughput sequencing. The *16S* sequences of *R. solanacearum* strains R31 and GMI1000 were compared to the OTU (operational taxonomic unit) representative sequences obtained from microbial group analysis using BLAST. Based on the results, the R31 strain was determined to be OTU000251 in the microbial group, while the *R. solanacearum* GMI1000 strain was determined to be OTU001444.

The Uparse algorithm (Uparse v7.0.1001) was employed to cluster effective tags across all samples (Edgar, 2013). Default parameters were used to cluster sequences into OTUs based on 97% identity. The representative sequences of each OTU were selected based on the highest frequency for annotation with species names using the Mothur method and the SILVA138 database (threshold set to 0.8–1). QIIME2 was used to compute α-diversity indices (Hall and Beiko, 2018), including observed species richness and Shannon indices for diversity. The visualizations of these indices were generated in R software (version 3.6.3). Differences among groups were determined using one-way ANOVA, with statistical significance set at *p* < 0.05. Additionally, the VEGAN package (version 2.5-7) in R was used to visualize the differences in taxonomic diversity among samples. Principal coordinate analysis (PCoA) was performed utilizing a Bray-Curtis distance matrix of taxon relative abundances (Pannaraj et al., 2017). To identify biomarkers among the different groups, linear discriminant analysis (LDA) effect size was conducted at the genus level. The screening threshold for differentially abundant microbiota was set to an LDA score > 2. Furthermore, the interactions among different microbiota were estimated at the genus level by calculating pairwise associations using the Spearman correlation coefficient. Only connections that met the criteria of *p*-value <0.01 and an absolute correlation value >0.6 were retained. Finally, the microbiome interaction networks were visualized using Cytoscape version 3.8.2 (Li et al., 2022).

### Statistical analysis

Unless stated otherwise, each experiment was conducted with a minimum of three biological replicates, and representative results are presented. Group similarity was assessed using Levene’s test and tested for normality using the Shapiro–Wilk test. Statistical analysis was carried out using Microsoft Excel (Office 365) and GraphPad Prism (version 8.02). A significance level of *p* ≤ 0.05 was used. Duncan’s test, ANOVA, and *t*-tests were used to determine statistically significant differences between data sets.

### Data availability

The data supporting the findings of this study are included in the paper and Supplementary material. Reagents, plant and microbe materials, and datasets used, generated, and analyzed during this study can be obtained from the corresponding author upon request. The sequencing data mentioned in this study have been deposited in the SRA database of the NCBI under accession number PRJNA1000974. The source data for the figures are provided as Supplementary material.

## Results

### *Bacillus subtilis* R31 significantly reduces the incidence of tomato bacterial wilt in pot experiments

Due to the good results achieved in the prevention and control of banana wilt disease (Li et al., 2011; Zhang et al., 2016; Cheng et al., 2017; Zhang et al., 2017; Li et al., 2021), the use of biocontrol agents with R31 as the main component for agricultural disease prevention and control has continuously expanded. We investigated whether R31 has a control effect on tomato bacterial wilt.

**Figure 1:**
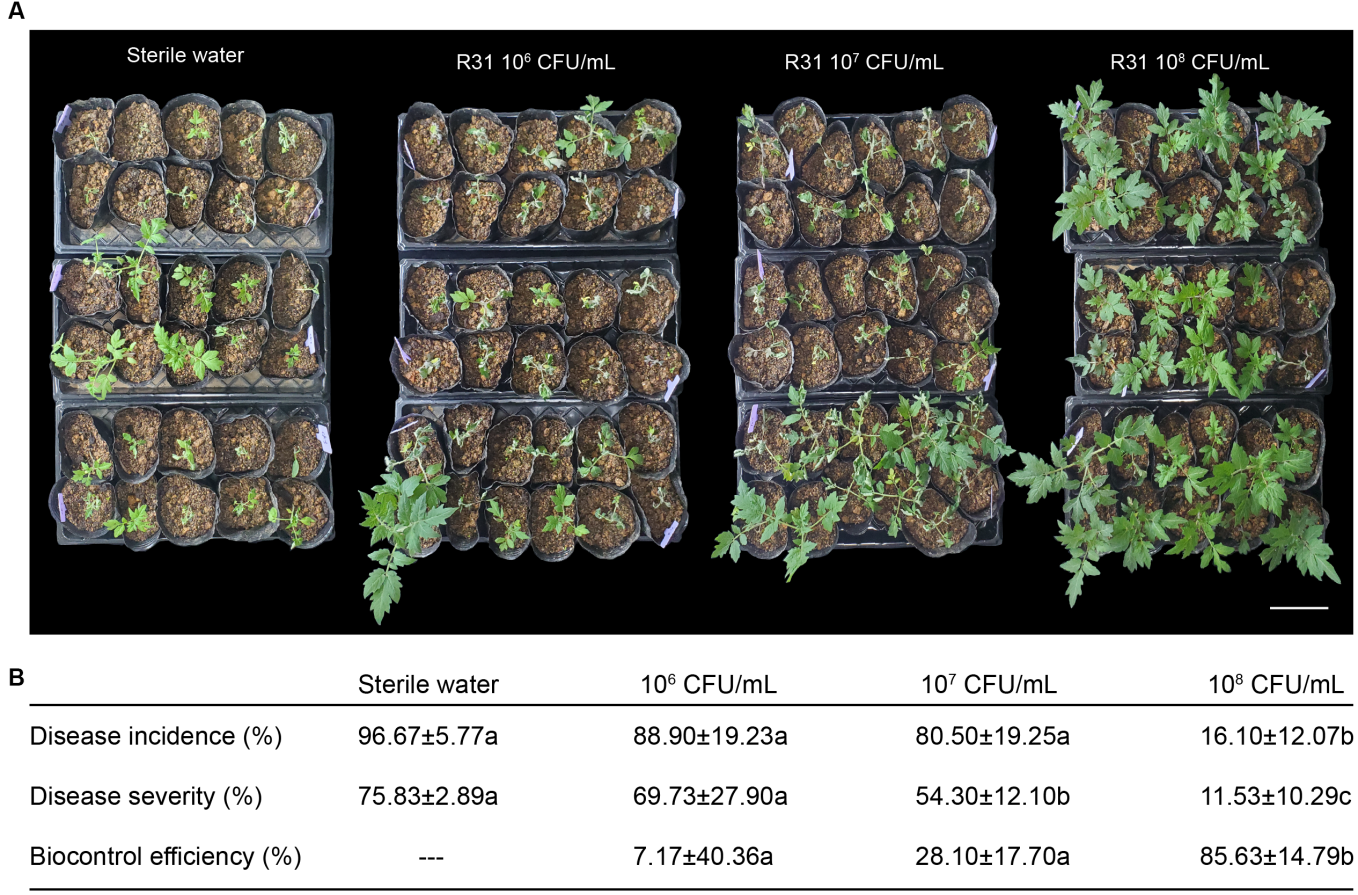
Pretreatment with *Bacillus subtilis* R31 reduces the incidence of tomato bacterial wilt. **(A)** Rhizosphere soil of 4-week-old tomato plants was inoculated with 50 mL of different titers of R31 fermentation broth or with 50 mL sterile water as the control. On the fifth day after R31 treatment, the rhizosphere soil of all plants was inoculated with 50 mL of 10^7^ CFU/mL *Ralstonia solanacearum* suspension. Photographs were taken on the 7th day after inoculation with *R. solanacearum*. **(B)** Disease incidence, disease severity, and biocontrol efficiency. Data are means ± standard deviation (SD, *n* ≥ 30). Different lowercase letters in the same column represent significant differences at *p* < 0.05 by ANOVA followed by Duncan’s test. Three biological replicates were conducted. Bar = 15 cm.

As shown in Figure 1A, we infused different titers of R31 fermentation broth into the rhizosphere soil of 4-week-old tomato plants at 5 days before inoculation with *R. solanacearum*. We then inoculated the plants with 10^7^ CFU/mL *R. solanacearum* cell suspension using the root injury method and scored disease incidence, disease severity, and biological efficiency 7 days after infection. Tomato plants showed varying degrees of disease occurrence in different experimental groups, with reduced disease severity observed in plants inoculated with R31 (Figure 1A). As shown in Figure 1B and Supplementary Table S1, the control plants (treated with sterile water only) had a disease incidence rate of 96.7% and a disease index of 75.83. However, the incidence rate of plants treated with strain R31 for early protection decreased significantly, and the control effect increased with increasing concentration of R31 during inoculation. The incidence rate and disease index of plants treated with R31 fermentation broth at a concentration of 10^8^ CFU/mL decreased to 16.1% and 11.5%, respectively, and the control effect increased to 85.6%. These results indicate that using R31 fermentation broth to protect the soil at the roots of plants in advance could significantly prevent and control the occurrence of tomato bacterial wilt.

### R31 colonizes tomato plant rhizosphere soil and roots while decreasing hydrogen peroxide and nitric oxide contents in root tissue

**Figure 2:**
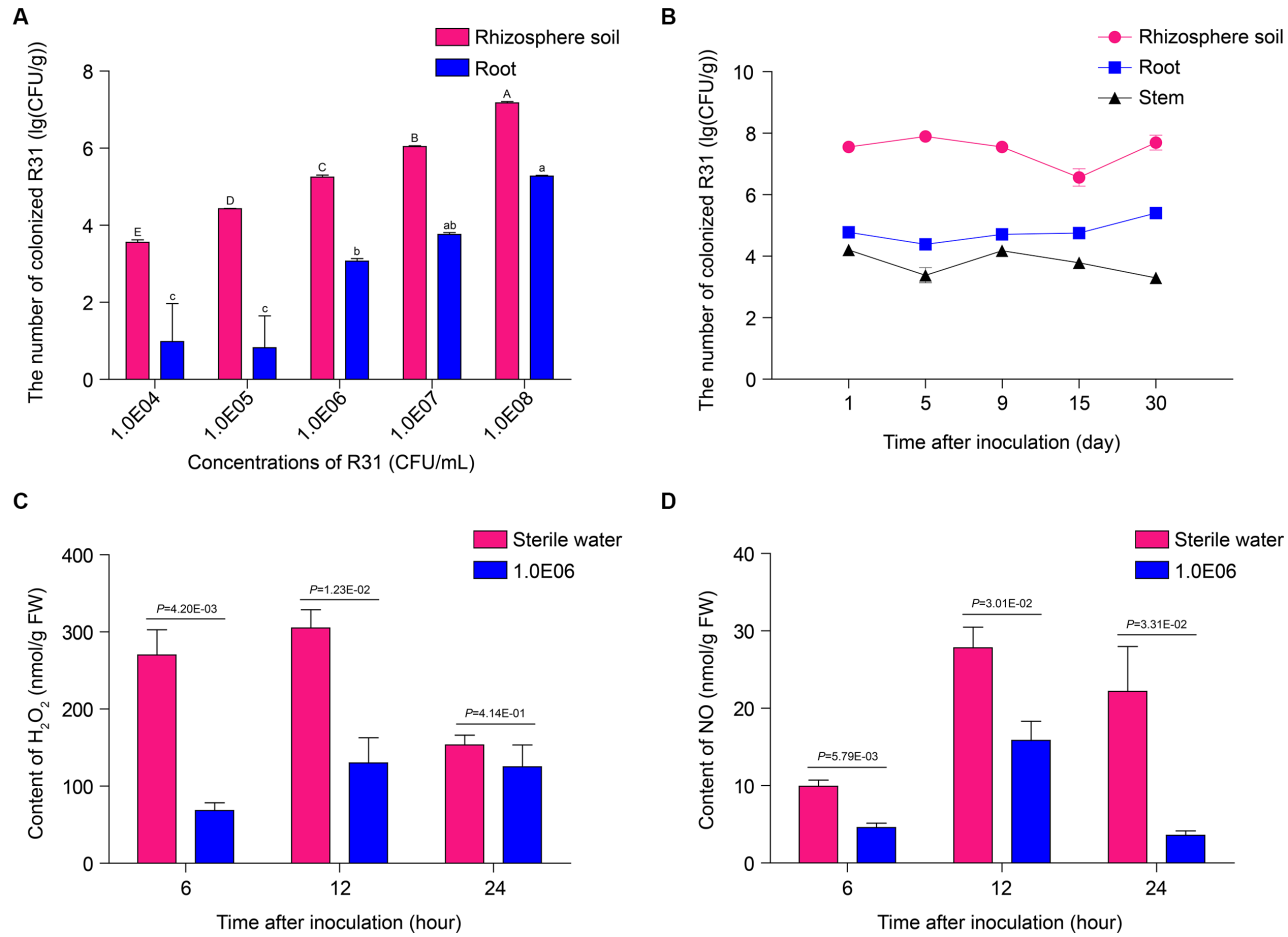
R31 forms stable colonies in soil and plant tissues and reduces reactive oxygen species contents in root tissues. **(A)** Colonization test of R31 in rhizosphere soil and roots of tomato. The rhizosphere soil of 4-week-old tomato plants was inoculated with 50 mL of different titers of R31 fermentation broth. On the 7th day after inoculation, live R31 cells in the rhizosphere soil and roots of tomato were counted. Data are means ± SD. Different lowercase or uppercase letters represent significant differences at *p* < 0.05 by Duncan’s test. Three biological replicates were conducted. **(B)** Colonization stability test of R31 in rhizosphere soil, roots, and stem tissues of tomato. The rhizosphere soil of 4-week-old tomato plants was inoculated with 50 mL of 10^8^ CFU/mL R31 fermentation broth. On the indicated days after inoculation, live R31 cells in rhizosphere soil and tissues of tomato were counted. Data are means ± SD. Three biological replicates were conducted. **(C,D)** The rhizosphere soil of 4-week-old tomato plants was inoculated with 50 mL of 10^6^ CFU/mL R31 fermentation broth; an equal volume of sterile water was used for the control. At the indicated time points after inoculation, H_2_O_2_ **(C)** and NO **(D)** contents in root tissues were measured. Data are means ± SD. *p-*values were determined by Duncan’s test. Three biological replicates were conducted.

We originally isolated R31, a strain with endophytic ability in plant tissues, from inside plant tissues (Li et al., 2011; Zhang et al., 2016; Cheng et al., 2017; Zhang et al., 2017; Li et al., 2021). The colonization ability of biocontrol bacteria is used as an indicator for evaluating their control effectiveness. To investigate whether R31 can form colonies in different biological environments during the prevention and control of tomato bacterial wilt, we measured the number of live bacteria in the soil, root tissue, and stems at different time points after inoculating R31 into the rhizosphere soil. At 7 days post inoculation, the number of live R31 cells in the rhizosphere soil and root tissue increased with increasing concentration of R31 during inoculation (Figure 2A; Supplementary Table S2). At different time points after inoculation with 10^8^ CFU/mL R31 cell suspension, as shown in Figure 2B and Supplementary Table S2, relatively stable concentration of R31 were still maintained in the rhizosphere soil, root tissue, and stem from day 1 to day 30.

Numerous studies have shown that *B. subtilis* colonies in rhizosphere soil or root tissue alter the host immune response, with an important indicator being ROS contents (Kotchoni and Gachomo, 2006; Sewelam et al., 2016; Sun et al., 2022). In this study, we investigated the levels of hydrogen peroxide and nitric oxide in the root tissues of plants at different time points after inoculation with 10^6^ CFU/mL R31 suspension. As shown in Figures 2C,D and Supplementary Table S2, compared to the control treatment, hydrogen peroxide contents significantly decreased in root tissue at 6 and 12 h of R31 treatment. In addition, the nitric oxide contents significantly decreased at 6, 12, and 24 h of R31 treatment. These results indicate that R31 can colonize tomato rhizosphere soil and roots to high titers and maintain long-term stability. In addition, R31 can lower the hydrogen peroxide and nitric oxide contents in tomato root tissue.

### R31 directly inhibits the growth of *Ralstonia solanacearum*

**Figure 3:**
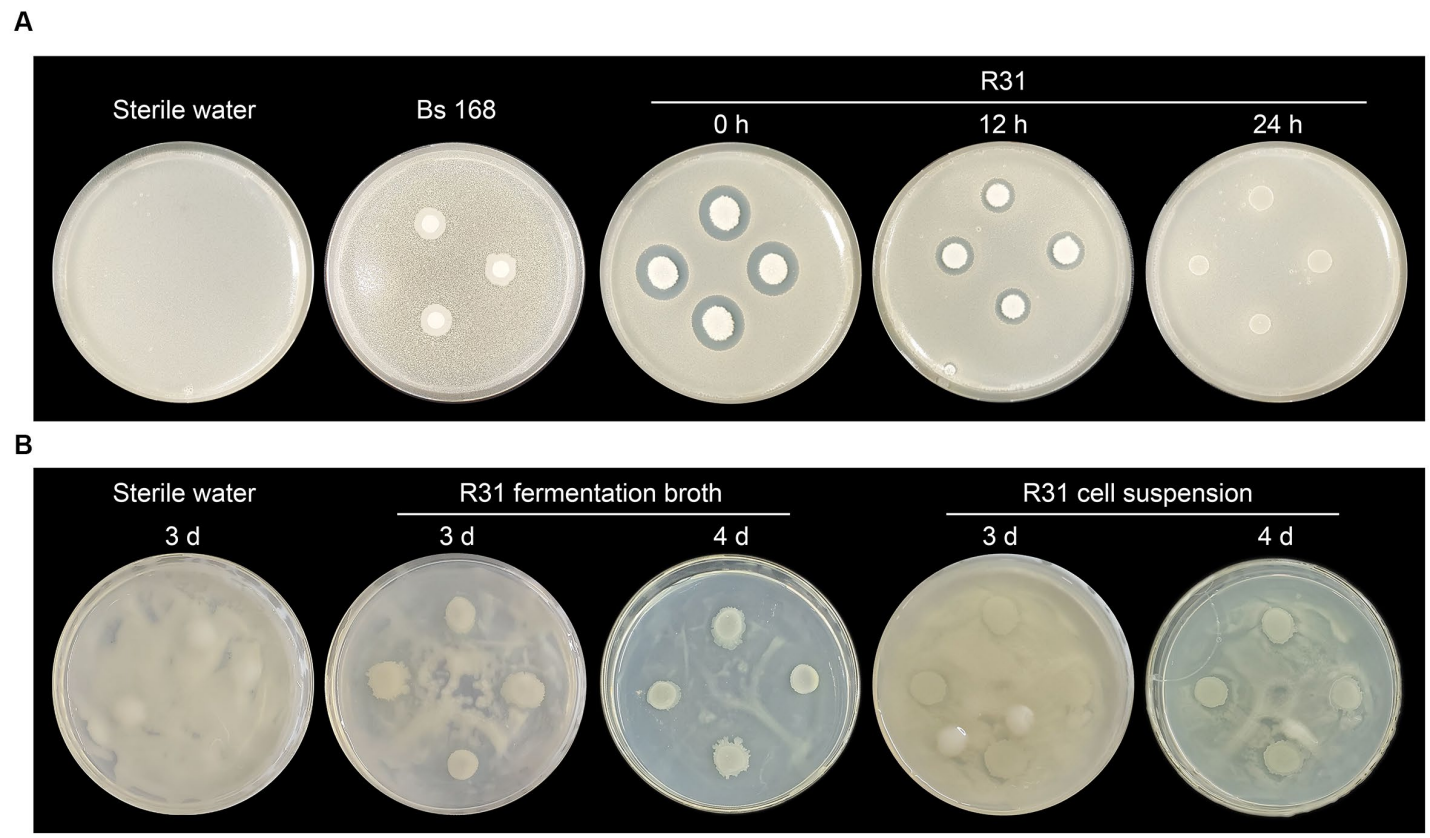
R31 directly inhibits the growth of *Ralstonia solanacearum*. **(A)** *Ralstonia solanacearum* (0.5 mL of 5 × 10^7^ CFU/mL) was mixed with 20 mL of cooled CPG agar medium prior to solidification. After the medium solidified, sterile filter paper disks were placed on the medium. The plates were incubated at 30°C for 0, 12, or 14 h, and 10 μL of 1 × 10^8^ CFU/mL *Bacillus subtilis* R31 fermentation broth was dropped onto the disks. The plates were incubated at 30°C for 3 days and photographed. Sterile water or *B. subtilis* 168 was dropped onto the filter paper disks as controls. **(B)** *Ralstonia solanacearum* (100 μL of 1 × 10^8^ CFU/mL) was spread evenly on the surface of a CPG agar medium plate. *Bacillus subtilis* R31 fermentation broth (10 μL of 1 × 10^8^ CFU/mL), or R31 bacterial suspension (10 μL of 1 × 10^8^ CFU/mL), prepared by washing cells with sterile water twice and resuspending in sterile water, was dropped onto the plate. The plates were incubated at 30°C for 3 to 4 days. Representative photographs are shown.

We previously demonstrated that R31 significantly inhibits the growth of plant pathogenic fungi (Li et al., 2011; Zhang et al., 2016; Cheng et al., 2017; Zhang et al., 2017; Li et al., 2021). To investigate whether R31 directly inhibits the growth of *R. solanacearum*, we conducted a plate confrontation experiment. First, we mixed *R. solanacearum* into agar medium. At different time points of culture of *R. solanacearum*, we dropped *B. subtilis* strains 168 and R31 onto the culture medium. Adding R31 immediately without preculturing *R. solanacearum* inhibited the growth of *R. solanacearum*. However, strain 168, a type strain of *B. subtilis* (Itaya, 2022), did not directly inhibit the growth of *R. solanacearum* (Figure 3A). Notably, when R31 was added after 12 h of *R. solanacearum* culture, the inhibitory effect of this strain on *R. solanacearum* growth was significantly diminished. Furthermore, when R31 was added after 24 h of *R. solanacearum* culture, we observed no growth inhibition (Figure 3A). These results suggest that it takes time for R31 to produce the specific substances that inhibit *R. solanacearum* growth.

To test this hypothesis, we directly coated the surface of the culture medium with *R. solanacearum* suspension and immediately added R31 fermentation suspension or washed R31 live cells. As shown in Figure 3B, in this more direct contact experiment, the fermentation broth of R31 produced clear antibacterial zones after 4 days of cultivation, whereas the radius of each antibacterial zone did not significantly decrease in the experimental group lacking the fermentation broth suspension components. These results indicate that R31 directly inhibits the growth of *R. solanacearum* and that this inhibition may require R31 to accumulate specific substances.

### R31 produces specific lipopeptide substances that directly inhibit the growth of *Ralstonia solanacearum* and the formation of *Ralstonia solanacearum* biofilm

**Figure 4:**
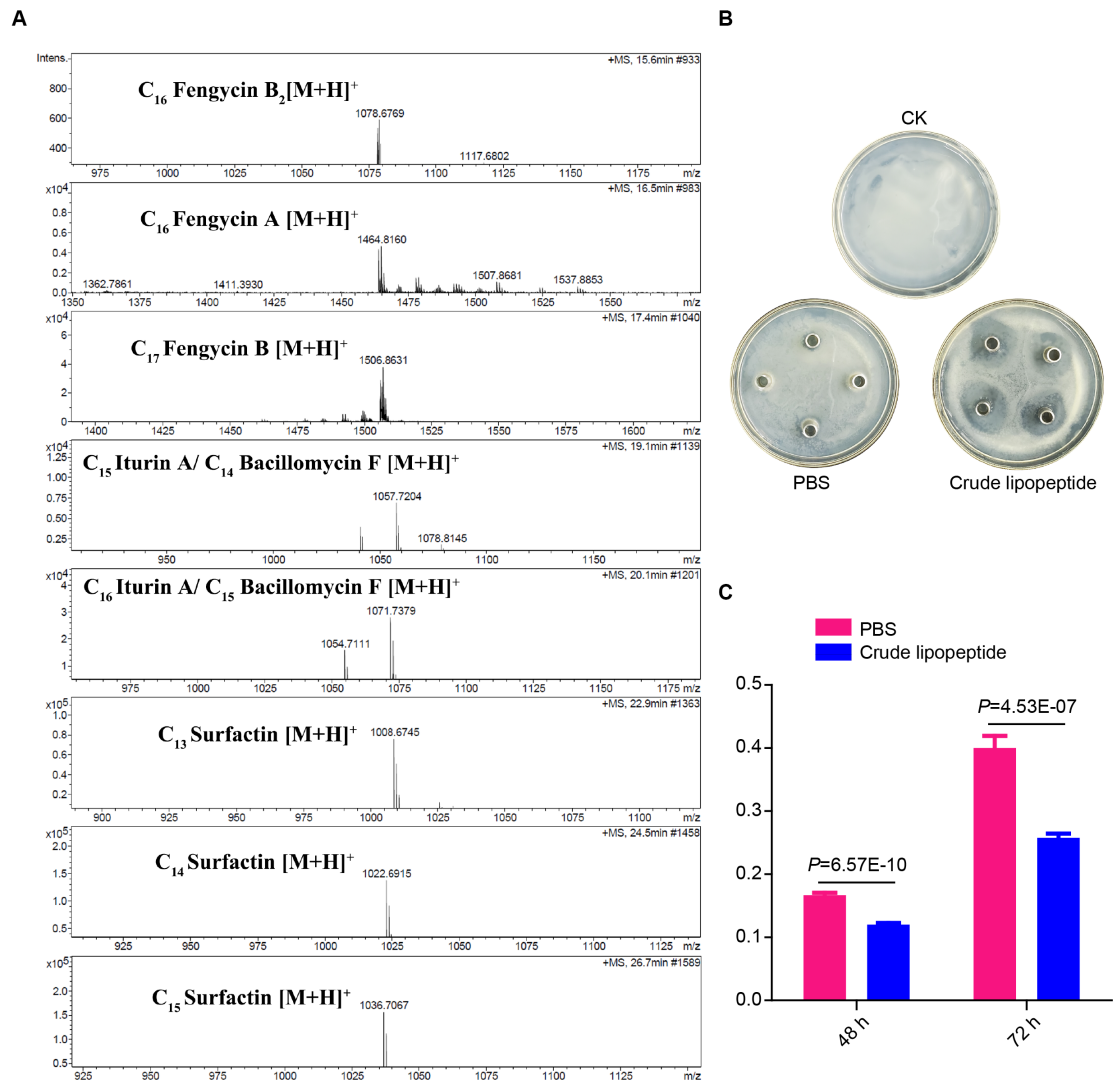
Lipopeptide substances produced by R31 directly inhibit the growth of *Ralstonia solanacearum* and the formation of *R. solanacearum* biofilm. **(A)** Mass spectrometry of crude lipopeptides produced by *Bacillus subtilis* R31. The peaks of eight detected lipopeptide substances are shown. **(B)** *Ralstonia solanacearum* cell suspension (100 μL of 1 × 10^6^ CFU/mL) was evenly plated onto CPG agar medium plate. Sterile Oxford cups were placed onto the plate, and 100 μL crude lipopeptide extract from R31 was added to each cup; an equal volume of phosphate-buffered saline (PBS) was used as the control. The plates were incubated at 30°C for 4 days. Representative photographs are shown. **(C)** CPG liquid medium (1,980 μL) and *R. solanacearum* cell suspension (10 μL of 10^8^ CFU/mL) were added to a 24-well cell culture plate and mixed with 10 μL of crude lipopeptide extract; PBS was used for the control. After 48 or 72 h of culture, the amount of *R. solanacearum* biofilm was measured by crystal violet staining. The absorbance values of each sample at 570 nm are shown. Data are means ± SD. *p-*values were determined by Duncan’s test.

To gain a deeper understanding of the biological characteristics of R31 and elucidate its biocontrol mechanism, we conducted whole-genome sequencing of R31 (Li et al., 2021). Many *B. subtilis* strains inhibit the growth of pathogens by secreting lipopeptides (Ben Abdallah et al., 2015; Cochrane and Vederas, 2016; Chen et al., 2019). To explore whether R31 inhibits the growth of *R. solanacearum* by producing lipopeptides, we identified several gene clusters synthesizing representative lipopeptides in the R31 genome (Supplementary Figure S1; Supplementary Table S3). To detect lipopeptide substances produced by R31, lipopeptides in R31 fermentation broth were sought by using ultra-high-resolution liquid chromatography–mass spectrometry. As shown in Figure 4A, we detected eight known lipopeptide compounds, with three from the fengycin family, two from the iturin family, and three from the surfactin family. In an Oxford cup experiment, as shown in Figure 4B, R31 crude lipopeptide extract successfully inhibited the growth of *R. solanacearum*. In addition, compared to solvents, crude lipopeptide extract significantly inhibited the production of *R. solanacearum* biofilm within 2–3 days of liquid culture (Figure 4C; Supplementary Table S4). These results indicate that R31 produces specific lipopeptide substances that directly inhibit the growth of *R. solanacearum* and the formation of *R. solanacearum* biofilm.

### R31 reduces *Ralstonia solanacearum* contents in rhizosphere soil and alters the composition of rhizosphere microorganisms

**Figure 5:**
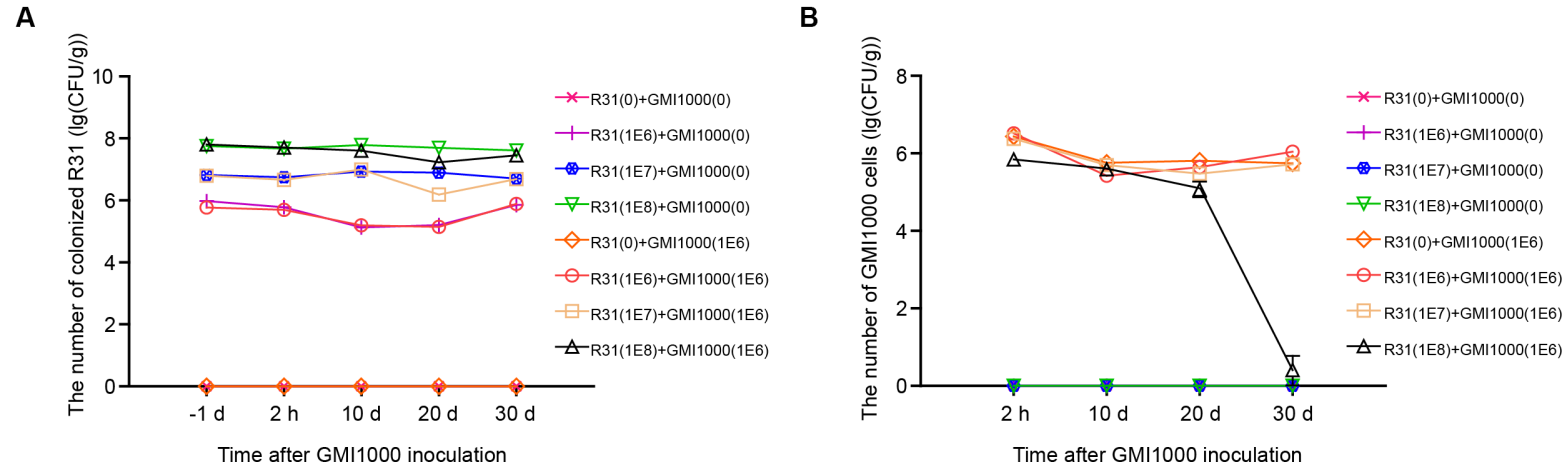
The mutual effects of R31 and *Ralstonia solanacearum* colonization in rhizosphere soil. The effect of *R. solanacearum* on the colonization of R31 in rhizosphere soil. Fifty milliliters of the indicated titer of R31 suspension was used to inoculate the rhizosphere soil, and an equal volume of the indicated titer of *R. solanacearum* was added to the soil at 6 days after R31 inoculation. The values in parentheses indicate the titer of bacterial suspension; 0 indicates an equal volume of sterile water as the control. Samples of rhizosphere soil from each plant were collected at the indicated time point after *R. solanacearum* inoculation. The quantity of R31 in rhizosphere soil was determined using the plate culture counting method **(A)**. Titer of *R. solanacearum* in rhizosphere soil as determined using qPCR **(B)**. Data are means ± SD.

We further investigated the relationship between R31 and *R. solanacearum* in the rhizosphere soil. As shown in Figure 5, we inoculated rhizosphere soil with different titers of R31 suspension, followed by inoculation with different titers of *R. solanacearum* suspension at 6 days after R31 inoculation. We measured the contents of R31 in rhizosphere soil at different time points after inoculation, finding that the R31 content in rhizosphere soil is positively correlated with the initial amount of R31 used during inoculation and is not affected by the inoculation with *R. solanacearum* (Figure 5A; Supplementary Table S5). However, *R. solanacearum* contents in the rhizosphere soil of the experimental group inoculated with R31 suspension in advance decreased compared to the control, which was not inoculated with R31. Furthermore, at 30 days after inoculation with 10^8^ CFU/mL R31, the *R. solanacearum* content rapidly decreased, and *R. solanacearum* was no longer detectable in some experimental groups (Figure 5B; Supplementary Table S5).

**Figure 6:**
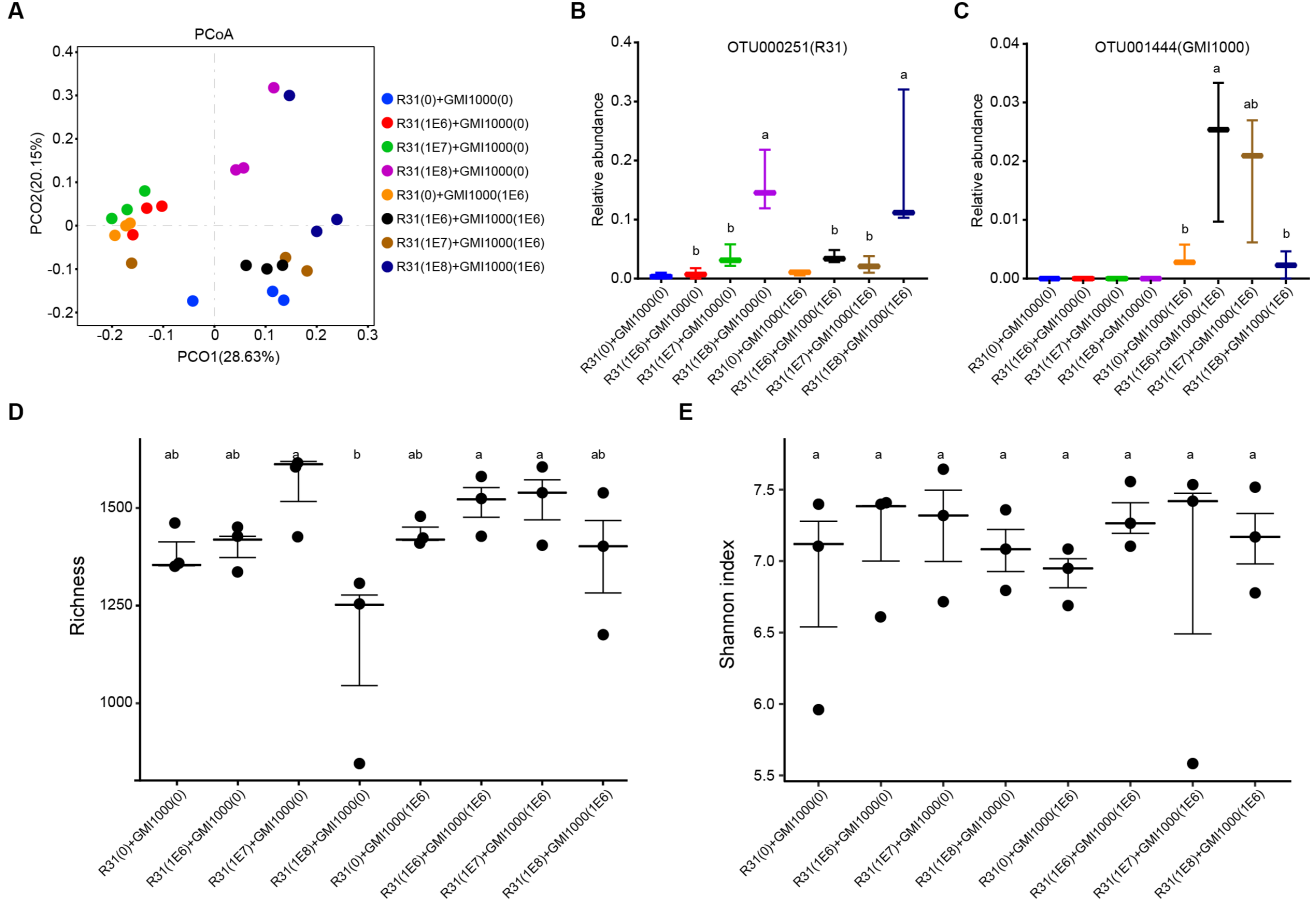
Effects of R31 and *Ralstonia solanacearum* on the rhizosphere microbial community. Fifty milliliters of the indicated titer of R31 suspension was inoculated into the rhizosphere soil. At 6 days after inoculation, an equal volume of the indicated titer of *R. solanacearum* was added to the soil. The values in parentheses indicate the titer of bacterial suspension; 0 indicates an equal volume of sterile water as the control. Samples of rhizosphere soil from each plant were collected at 20 days after *R. solanacearum* inoculation. **(A)** Microbial communities for all groups based on the results of PcoA β diversity analysis. The different colored circles represent different values of rhizosphere soil after adding different combinations of various titers of R31 or *R. solanacearum* suspension. **(B,C)** Relative abundances of OTU000251 **(B)** and out001444 **(C)** in the indicated samples. OTU000251 represents R31, and OTU001444 represents *R. solanacearum*. **(D)** Richness and **(E)** Shannon indices between the corresponding soil samples. Different lowercase letters represent significant differences at *p* < 0.05 by Duncan’s test.

To investigate the influence of R31 on the microbial community in rhizosphere soil in the absence or presence of *R. solanacearum*, we examined the microbial composition in the rhizosphere soil of each plant at 20 days after treatment with different titers of R31 and *R. solanacearum*. As shown in Figure 6A, PcoA showed that the repeatability in each group was generally good, and different samples in the same group tended to aggregate together. We subjected the relative abundances of the OTUs corresponding to R31 and *R. solanacearum* strain GMI1000 to statistical analysis. In general, the relative abundances of R31 were positively correlated with the titers of the applied bacterial suspension (Figure 6B). The relative abundances of *R. solanacearum* in the presence of R31 significantly decreased with increasing R31 concentration used for treatment (Figure 6C), which is consistent with the results described above (Figures 2A, 5). We then analyzed the diversity in microbial community of each group. Except when adding high concentrations of R31 (10^8^ CFU/mL) alone, the other treatments had little effect on the richness of the microbial community (Figure 6D). However, there was no significant difference in the Shannon index among groups (Figure 6E). These results indicate that R31 significantly reduces the content of *R. solanacearum* in rhizosphere soil and alters the composition of rhizosphere microorganisms.

### Screening of interacting species between R31 and *Ralstonia solanacearum*

Correlation analysis between the relative abundances of R31 and *R. solanacearum* revealed a Spearman correlation coefficient of −0.45 (*p* = 0.22), pointing to an apparent negative correlation between the relative abundances of these two strains in soil; however, this correlation was not significant. Therefore, we reasoned that R31 might also play a role in controlling bacterial wilt by affecting other bacterial communities that coexist with *R. solanacearum*. We thus analyzed the potential interacting microbiota with R31 in the absence and presence of *R. solanacearum*. We selected bacterial genera for each group based on the thresholds “average relative abundance > 10^−5^” and “detected in ≥50% of samples.” We performed Spearman correlation analysis of the selected bacterial genera based on their relative abundance information. We identified significant correlations based on the thresholds “correlation coefficient R absolute value > 0.8 (extremely strong correlation)” and “*p* < 0.01.” In soil lacking *R. solanacearum*, R31 was positively correlated with *Sphingobacterium*, *Brevundimonas*, *Algoriphagus*, and *Bacteriovorax* and negatively correlated with bacteria such as *Dyella* and *Paenibacillus* (Supplementary Table S6). In soil containing *R. solanacearum*, R31 was positively correlated with *Peredibacter*, *Glutamicibacter*, *Sphingobacterium*, *Ochrobactrum*, *Stenotrophomonas*, and *Bdellovibrio* and negatively correlated with *Curvibacter* and *Sphaerobacter* (Supplementary Table S7). However, *R. solanacearum* was negatively correlated with *Acidovorax*, and it was associated with *Methylophilus*, *Dyella*, *Azospirillum*, *Nocardioides*, *Sphingomonas*, *Lysinibacillus, Candidatus* Jidaibacter, and *Cavicella*. Since the genera directly related to R31 do not intersect with the genera directly related to *R. solanacearum*, we further analyzed the association between R31 and *R. solanacearum* by examining the interactions of R31 with other genera, as described below.

### Construction of a microbial interaction network and functional analysis of the microbial community for R31-induced inhibition of *Ralstonia solanacearum*

**Figure 7:**
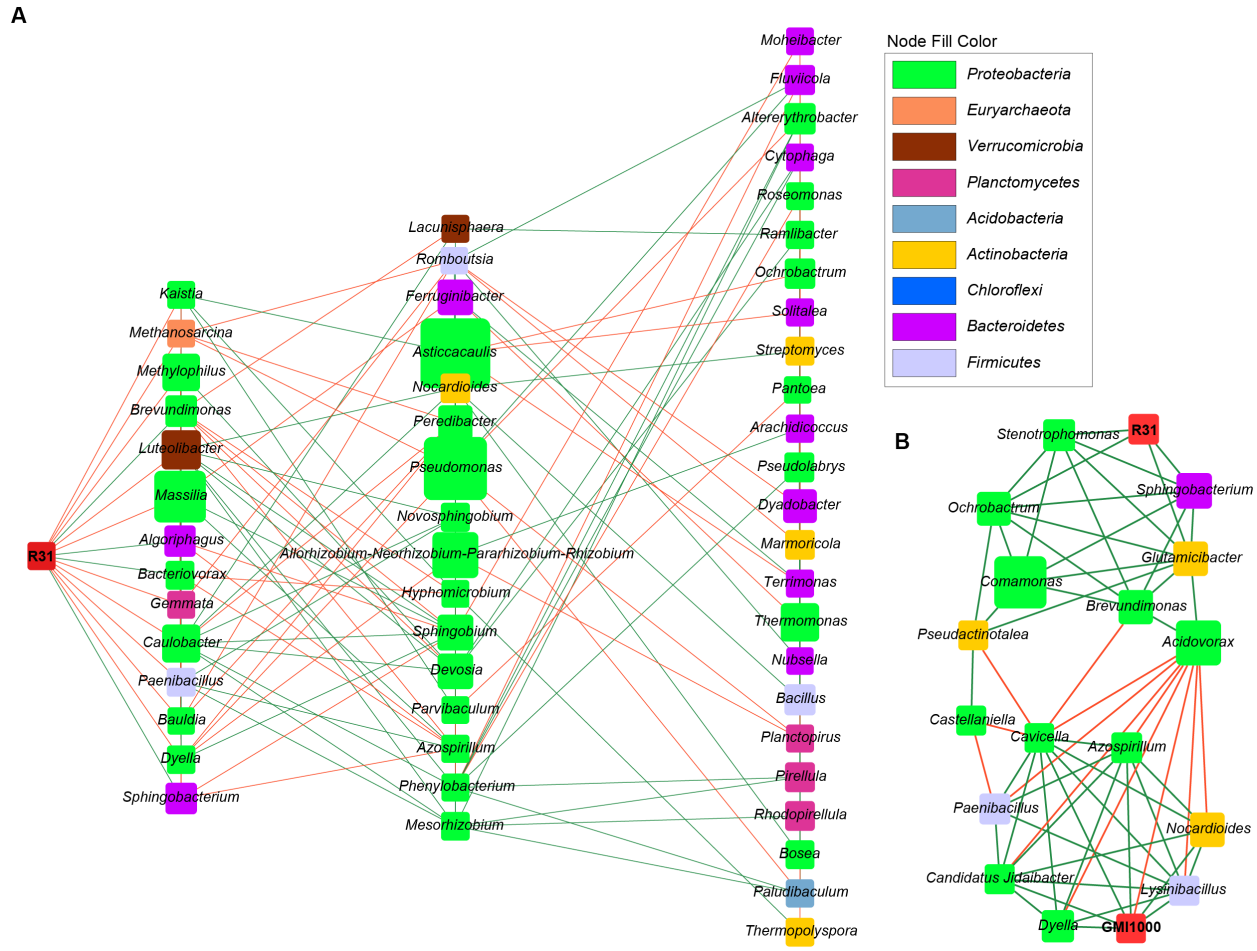
The microbial interaction network of R31 and *Ralstonia solanacearum* in rhizosphere soil. **(A)** The microbial community interaction network of R31 in rhizosphere soil in the absence of *R. solanacearum*. **(B)** The R31-regulated microbial community that interacts with *R. solanacearum* GMI1000. The node size represents the relative abundance of species, and different colors represent different phylum classifications. Green lines indicate positive correlations, and orange lines indicate negative correlations.

Using Cytoscape software, we constructed the microbial community interaction network of R31 in soil in the absence *R. solanacearum* (Figure 7A) and the microbial community regulatory network of R31 with *R. solanacearum* (Figure 7B). Comparing the two network diagrams, we determined that four genera, *Sphingobacterium*, *Brevundimonas*, *Dyella*, and *Paenibacillus*, directly interact with R31 in soil in the absence of *R. solanacearum*. These genera also interacted (directly or indirectly) with R31 in soil containing *R. solanacearum*; these interactions are consistent with those observed in soil in the absence of *R. solanacearum*. R31 positively regulates *Acidovorax* abundance via *Glutamicibacter*, and *Acidovorax* and *R. solanacearum* are mutually exclusive (Figure 7B). R31 also negatively regulates the abundances of bacterial genera that coexist with *R. solanacearum* by indirectly regulating the abundances of three genera: *Pseudoactinotalea*, *Pseudoactinotalea*, and *Brevundimonas*.

**Figure 8:**
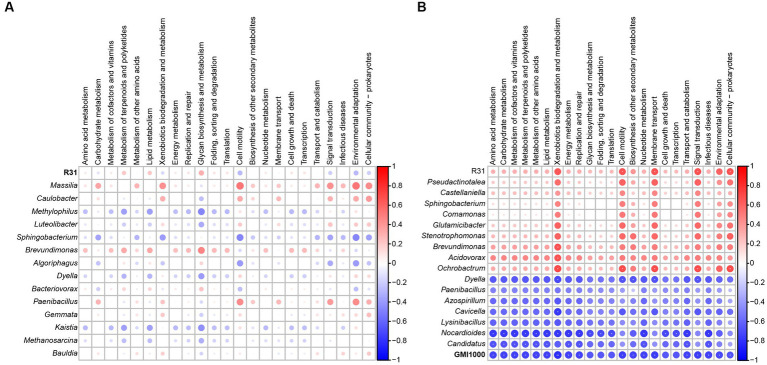
Correlation analysis of functional pathways between R31, *Ralstonia solanacearum*, and related genera and microbiota. **(A)** Correlation analysis of functional pathways between R31 and related genera and microbial communities in rhizosphere soil in the absence of *R. solanacearum*. **(B)** Correlation analysis of functional pathways between related genera and microbial communities in rhizosphere soil with interactions between R31 and *R. solanacearum*. ^**^p < 0.01; ^*^0.01 ≤ *p* < 0.05.

Based on the relative abundance of each species in different samples in the interaction network, as well as the abundance information of different functional pathways predicted by Taxa4Fun software in each sample, we performed a Spearman correlation analysis to predict the role of each species in the structures and functions of microbial communities. In soil lacking *R. solanacearum* with only R31 added, the effect of R31 on bacterial community function was not significant, and other bacterial communities that directly interacted with R31 also had no significant influence on community function (Figure 8A). In soil where R31 and *R. solanacearum* were added simultaneously, the effects of R31 and *R. solanacearum* on bacterial community function showed an opposite trend (Figure 8B). At the same time, the bacterial genera that were positively correlated with R31 also promoted bacterial community function as a whole, while the bacterial genera that were positively correlated with *R. solanacearum* had an inhibitory effect on bacterial community function.

## Discussion

The banana wilt pathogen is a eukaryote (Ploetz, 2015), and the *R. solanacearum* pathogen is a prokaryote. However, these two pathogens lead to typical soil-borne vascular bundle diseases (Ploetz, 2015), which might be an important reason why R31 has control effects on both diseases. We propose that R31 treatment has several advantages when dealing with soil-borne diseases of plant vascular bundles. Firstly, R31 has direct antibacterial activity. This strain can directly contact *Fusarium oxysporum* and *R. solanacearum* in the soil and significantly inhibit their growth, especially in the rhizosphere soil environment. Secondly, the colonization ability of R31 plays a role in maintaining the microbiota and regulating the duration of plant immunity. Finally, R31 has a significant ability to regulate microbial communities and has good ecological prevention and control capabilities for disease transmission caused by soil-borne pathogens.

The disease prevention and control effects of R31 showed highly significant dose-effect dependence on the initial R31 titer used for treatment (Figures 1, 5B, 6B,C). When a titer of 10^8^ CFU/mL R31 was applied, the disease control effect reached a very high level. However, this effect might be due to the inhibitory effect of R31 on pathogenic bacteria, which requires the accumulation of sufficient antibacterial substances (Figures 3, 4). Another possibility is that a sufficient titer of R31 might be required to efficiently and stably regulate the microbial community structure in microbial community interaction networks (Figures 7, 8).

The effective colonization of biocontrol bacteria in plants may help them exert their biological functions to a certain extent (Syed Ab Rahman et al., 2018; Vurukonda et al., 2018; Yan et al., 2019), but breaking through the front line of plant defense is also a problem that biocontrol bacteria need to overcome. Our findings indicate that R31 may help colonize the host tissue by decreasing the ROS contents at the invasion site (Figures 2C,D). However, lower ROS contents may lead to a decrease in the resistance of plant tissues to pathogen invasion. The specific adjustment mechanism requires further study in the future. Nonetheless, these results suggest that the early application of biocontrol bacteria such as R31 before disease onset may be crucial for ensuring that the bacteria first colonize the plant roots.

In the strain confrontation experiment on agar culture medium, R31 had a relatively significant effect on inhibiting the growth of *R. solanacearum* when we used the standard protocol (Figure 3). However, by altering the experimental conditions and protocol, the effect of R31 on inhibiting *R. solanacearum* growth significantly decreased when *R. solanacearum* was allowed to grow for a longer period of time or when the fermentation broth components of R31 were removed (Figure 3). These results suggest that when using R31 for disease prevention and control, it may be necessary to decrease the titer of pathogenic bacteria via sterilization before using R31 for disease prevention and control if the titer of pathogenic bacteria in the soil is high. There are also components in the culture medium of R31 that significantly inhibit *R. solanacearum* growth, indicating that under specific prevention and control scenarios, using formulations with fermentation broth might be more effective than using on-site dissolved bacterial powder formulations. In fact, the commercial microbial agent product “Diyixi” and the microbial fertilizer “Dingwei,” which contain R31 as their core component, are both liquid preparations.

The relative abundance of *R. solanacearum* gradually decreased with increasing titer of R31 (Figures 6B,C). However, correlation analysis suggested that these two bacteria may also indirectly interact with each other. Construction of the microbial community interaction network also indicated that R31 can indirectly decrease the abundance of *R. solanacearum* by regulating the abundances of multiple bacteria that co-occur with *R. solanacearum* (Figure 7). Notably, functional correlation analysis of bacterial communities showed that the abundance of *R. solanacearum* and its co-occurring bacterial communities was negatively correlated with the enrichment of metabolic functions in the entire soil microbial community, suggesting that *R. solanacearum* may inhibit the growth of most normal bacterial communities that contribute to metabolic functions in the soil (Figure 8). Adding R31 to the soil can indirectly inhibit the growth of *R. solanacearum* and its co-occurring bacterial communities, thereby restoring the abundances of other normal bacterial communities that contribute to metabolic function. In the absence of *R. solanacearum*, adding R31 alone has no significant effect on the function of the soil microbial community under laboratory conditions.

### Contributions of research findings

Bacterial wilt caused by *R. solanacearum* is a widespread, severe plant disease. Tomato, one of the most important vegetable crops worldwide, is particularly susceptible to this disease. The effectiveness of traditional prevention and control methods for this disease is very limited. Moreover, these methods pose significant issues such as environmental pollution and the accumulation of pesticide residues. Biological control offers numerous advantages over chemical control. In this study, we demonstrated that the early application of biological control strain *B. subtilis* R31 can significantly decrease the incidence rate of tomato bacterial wilt disease. In addition, R31 directly inhibits the growth of *R. solanacearum*, with an important role played by lipopeptides secreted by R31. Finally, we determined that R31 can stably colonize the rhizosphere soil and roots of tomato plants for a long time, lower the *R. solanacearum* content in the rhizosphere soil, and inhibit the growth of the microbial community that facilitates infection by *R. solanacearum*. This study provides an important theoretical basis for elucidating the mechanism of action of *B. subtilis* in biological control against bacterial wilt and lays the foundation for the optimization and promotion of other agents such as R31.

## Data availability statement

The datasets presented in this study can be found in online repositories. The names of the repository/repositories and accession number(s) can be found in the article/Supplementary material.

## Author contributions

YS: Conceptualization, Data curation, Formal analysis, Funding acquisition, Investigation, Methodology, Project administration, Resources, Software, Visualization, Writing – original draft, Writing – review & editing. YS: Conceptualization, Data curation, Formal analysis, Investigation, Methodology, Project administration, Software, Writing – original draft. ZM: Conceptualization, Data curation, Investigation, Methodology, Software, Writing – original draft. JZ: Formal analysis, Software, Visualization, Writing – review & editing. LZ: Conceptualization, Data curation, Formal analysis, Investigation, Methodology, Writing – original draft. SM: Data curation, Formal analysis, Software, Writing – original draft. DQ: Data curation, Investigation, Methodology, Writing – review & editing. YR: Formal analysis, Methodology, Software, Writing – original draft. YW: Data curation, Formal analysis, Writing – original draft, Writing – review & editing. LX: Conceptualization, Data curation, Formal analysis, Software, Writing – original draft. XY: Data curation, Formal analysis, Methodology, Writing – review & editing. ZD: Formal analysis, Methodology, Resources, Writing – review & editing. PC: Data curation, Validation, Visualization, Writing – review & editing. MS: Data curation, Formal analysis, Supervision, Writing – review & editing. GY: Resources, Supervision, Visualization, Writing – review & editing.
