# Supplementary material for: Biocontrol of bacterial wilt disease in tomato using Bacillus subtilis strain R31
Source: Front Microbiol. 2023 Sep 28;14:1281381. doi: 10.3389/fmicb.2023.1281381 (PMC10568012; doi:10.3389/fmicb.2023.1281381)
Supplement: Supplementary file 3 [file Table_3.DOCX]

Table S3. The predicted biosynthesis cluster of lipopeptide substances *B. subtilis* R31

| Predictive substances | Size (kb) |
| --- | --- |
| Fengycin | 77.112 |
| Bacillaene | 105.261 |
| Surfactin | 63.417 |
| Subtilomycin | 24.458 |
| Bacilysin | 41.419 |
| Subtilosin | 21.612 |
| Bacillibactin | 47.137 |
