# Supplementary figures and images for: Biocontrol of bacterial wilt disease in tomato using Bacillus subtilis strain R31
Source: Front Microbiol. 2023 Sep 28;14:1281381. doi: 10.3389/fmicb.2023.1281381 (PMC10568012; doi:10.3389/fmicb.2023.1281381)

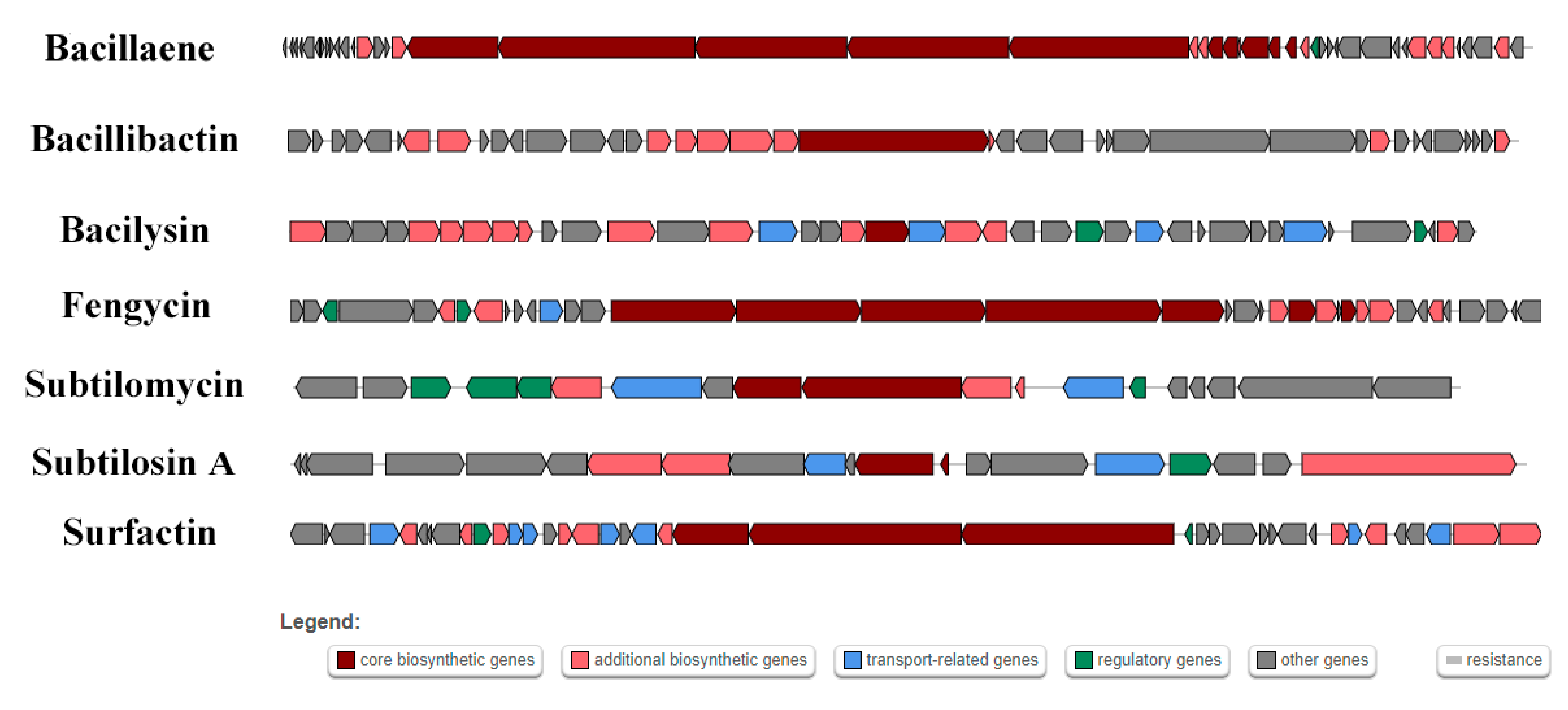

Supplement: Supplementary file 8 [file Image_1.TIF]
